# ASO Author Reflections: Similar Long-Term Survival After Transhiatal Compared to Transthoracic Esophagectomy

**DOI:** 10.1245/s10434-022-12443-x

**Published:** 2022-09-16

**Authors:** Anna Junttila, Olli Helminen, Joonas H. Kauppila, the FINEGO Group, Olli Helminen, Mika Helmiö, Heikki Huhta, Raija Kallio, Vesa Koivukangas, Arto Kokkola, Simo Laine, Elina Lietzen, Sanna Meriläinen, Vesa-Matti Pohjanen, Tuomo Rantanen, Ari Ristimäki, Jari V. Räsänen, Juha Saarnio, Eero Sihvo, Vesa Toikkanen, Tuula Tyrväinen, Antti Valtola

**Affiliations:** 1 Division of Digestive Surgery and Urology, Turku University Hospital, Turku, Finland; 2 Surgery Research Unit, Medical Research Center Oulu, Oulu University Hospital and University of Oulu, Oulu, Finland; 3 Upper Gastrointestinal Surgery, Department of Molecular Medicine and Surgery, Karolinska Insitutet and Karolinska University Hospital, Stockholm, Sweden

## Past

Transhiatal esophagectomy (THE) and transthoracic esophagectomy (TTE) are valid surgical procedures for esophageal cancer. TTE allows for more extensive lymphadenectomy via thoracic dissection, while THE has been preferred in frail patients, as some studies have shown higher morbidity and short-term mortality after TTE. Both of these procedures can also be performed with minimally invasive techniques. However, available evidence of long-term oncological outcomes after THE compared with TTE^1,2^ is contradictory, and there is also a lack of population-based studies. So far, existing studies do not show superiority of either approach in treatment of esophageal cancer, and the largest published meta-analysis of 52 studies found no difference in 5-year overall survival.^3^

## Present

We conducted a population-based, nationwide cohort study including all 1338 patients with esophageal cancer undergoing curatively intended THE and TTE in Finland from 1987 to 2016.^4^ We used Cox regression adjusted for confounders to investigate 5-year and 90-day survival, as well as explanatory subgroup analyses. The observed 5-year survival was 39.3% after THE and 45.0% after TTE, and 90-day observed survival was 89.5% versus 92.3%, respectively. However, no statistically significant differences were present in 5-year or 90-day observed survival comparing THE with TTE in any of the adjusted models, nor in the five subgroups examined.

## Future

The present study suggests similar 5-year survival after THE and TTE. Despite higher counts of examined lymph nodes in the transthoracic approach, no difference in long-term survival was present. The cervical anastomosis inherent to the THE technique has been associated with higher leak rates; however, leaks after TTE can be more severe due to mediastinal manifestations. In the current study, complication rates were not studied; however, 90-day mortality rates were similar after THE and TTE. Although it seems from this evidence that the surgical approach can be chosen on the basis of surgeon preference, further studies are needed, especially on certain subgroups such as tumors with high T-stage, tumors located in the middle third of the esophagus, those with lymph node metastases in thoracic nodes, and operations performed with minimally invasive techniques.
